# Supplementary material for: Strategic Optimization of the Middle Domain IIIA in RBP-Albumin IIIA-IB Fusion Protein to Enhance Productivity and Thermostability
Source: Int J Mol Sci. 2024 Dec 27;26(1):137. doi: 10.3390/ijms26010137 (PMC11720212; doi:10.3390/ijms26010137)

Supplementary Figure S1. Schematic representation of the fusion protein R31. The diagram shows R31, consisting of the retinol-binding protein (RBP) and albumin domains IIIA and IB, alongside full-length albumin and RBP for comparison. The fusion protein is tagged with histidine residues at the C-terminal, with amino acid positions indicated by numbers.

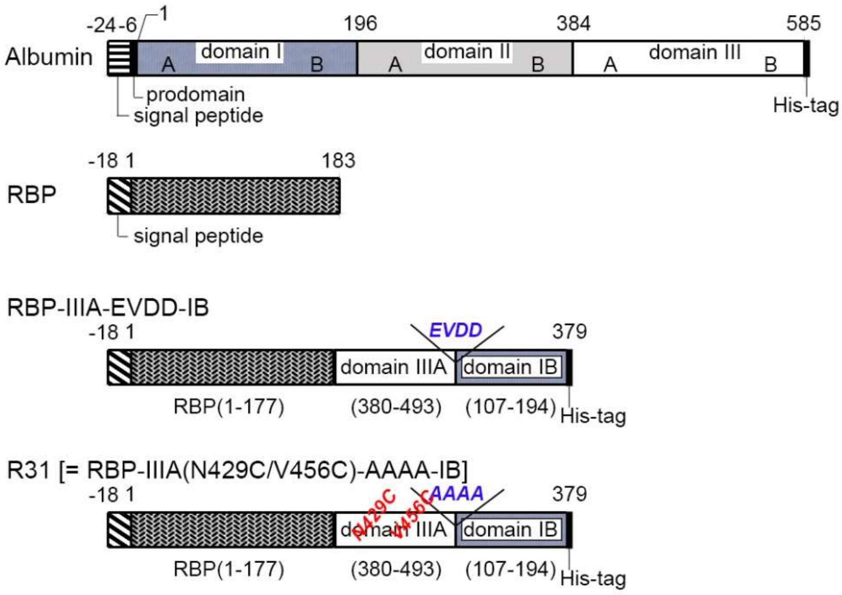

Amino acid sequence

RBP-IIIA-EVDD-IB	ERDCRVSSFRVKENFDKARFSGTWYAMAKKDPEGLFLQDNIVAEFSVDETGQMSATAKGRVRL<br>LNNWDVCADMVGTFTDTEDPAKFKMKYWGVASFLQKGNDDHWIVDTDYDTYAVQYSC<br>RLLNLDGTCADSYSFVFSRDPNGLPPEAQKIVRQRQEELCLARQYRLIVHNGYCDGRLVE<br>EPQNLIKQNCELFEQLGEYKFQNALLVRYTKKVPQVSTPTLVEVSR**N**LGKVGSKCCKHPE<br>AKRMPCAEDYLSV**V**LNQLCVLHEKTPVSDRVTKCCTESLVNRRPCFSAL**EVDD**NPNLPRL<br>VRPEVDVMCTAFHDNEETFLKKYLYEIARRHPYFYAPELLFFAKRYKAAFTECCQAADKA<br>ACLLPKLDELRDEGKASSA
R31 [= RBP-IIIA(N227C/V254C)-AAAA-IB]	ERDCRVSSFRVKENFDKARFSGTWYAMAKKDPEGLFLQDNIVAEFSVDETGQMSATAKGR<br>VRLLNNWDVCADMVGTFTDTEDPAKFKMKYWGVASFLQKGNDDHWIVDTDYDTYAVQYSC<br>RLLNLDGTCADSYSFVFSRDPNGLPPEAQKIVRQRQEELCLARQYRLIVHNGYCDGRLVE<br>EPQNLIKQNCELFEQLGEYKFQNALLVRYTKKVPQVSTPTLVEVSR**C**LGKVGSKCCKHPE<br>AKRMPCAEDYLSV**C**LNQLCVLHEKTPVSDRVTKCCTESLVNRRPCFSAL**AAAA**NPNLPRL<br>VRPEVDVMCTAFHDNEETFLKKYLYEIARRHPYFYAPELLFFAKRYKAAFTECCQAADKA<br>ACLLPKLDELRDEGKASSA

Supplementary Figure S2. The predicted structures of R31 (**A**) and R31-G176K (**B**) generated using AlphaFold2. In these models, the RBP domain is shown in orange and the albumin domains IIIA and IB are depicted in cyan and magenta, respectively.

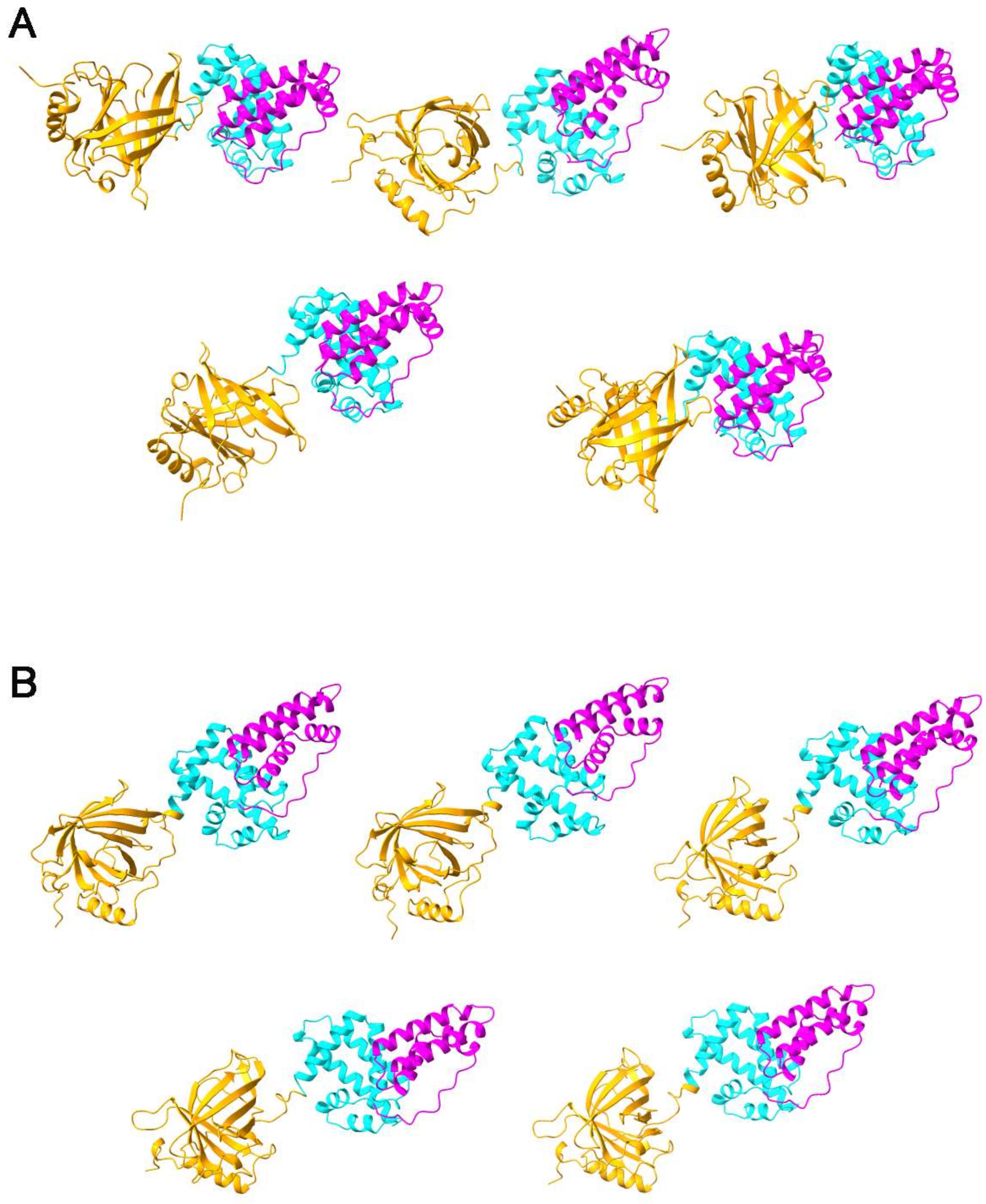

Supplement: Supplementary file 1 [file ijms-26-00137-s001.zip › ijms-3370831-supplementary.pdf]
